# Household and family factors related to weight status in first through third graders: a cross-sectional study in Eastern Massachusetts

**DOI:** 10.1186/1471-2431-14-167

**Published:** 2014-07-01

**Authors:** Sonya Irish Hauser, Christina D Economos, Miriam E Nelson, Jeanne P Goldberg, Raymond R Hyatt, Elena N Naumova, Sarah E Anderson, Aviva Must

**Affiliations:** 1 The Sage Colleges, School of Health Sciences, 65 First St, Troy, NY 12180, USA; 2 Gerald J. and Dorothy R. Friedman School of Nutrition Science and Policy, Tufts University, 150 Harrison Avenue, Boston, MA 02111, USA; 3 Department of Public Health and Community Medicine, Tufts University School of Medicine, 136 Harrison Avenue, Boston, MA 02111, USA; 4 School of Engineering, Tufts University, 200 College Ave, Medford, MA 02155, USA; 5 Division of Epidemiology, College of Public Health, The Ohio State University, 1841 Neil Avenue, Columbus, Ohio 43210, USA

**Keywords:** Childhood obesity, Prevention, Family dinner, Household environment

## Abstract

**Background:**

Early environmental influences have been linked to child weight status, however further understanding of associations in diverse populations is needed.

**Methods:**

A cross-sectional analysis of household and family factors associated with overweight was conducted on a culturally diverse, urban dwelling sample of 820 first through third graders (mean age 7.6 ± 1.0 years) residing in three eastern Massachusetts cities. Overweight was defined as BMI > 85th percentile, based on measured height and weight, and the CDC growth reference. Multivariate logistic regression was used to identify demographic, behavioral, and social environmental variables significantly related to weight status. Independent variables included race-ethnicity, age, sex, servings of sugar-sweetened beverages/week, hours of screen time/week, parent overweight, (calculated from self-reported weight/height), parent education, household food restriction rules regarding snacking and/or kitchen access, frequency of having dinner as a family (reported as “a lot” vs. “sometimes/rarely/never”) and child vitamin/mineral supplement use. Selected interactions were explored based on prior studies.

**Results:**

Prevalence of overweight was 35.5% in girls and 40.8% in boys. In the final, adjusted model, compared to white children, the odds of overweight were higher in children of Hispanic race-ethnicity (odds ratio (OR) = 2.4, 95% CI = 1.4 - 4.1). In the same adjusted model, compared to children with no household food restriction rules, the odds of overweight were 2.6 (95% CI = 1.3-5.1) times higher and 3.5 (95% CI = 1.9-6.4) times higher for children having one rule or two rules, respectively. Parent report of frequent family dinner and child vitamin use were protective, with a halving of risk for overweight for each behavior (OR = 0.47, 95% CI = 0.31-0.71 and OR = 0.54, 95% CI = 0.37-0.78, respectively).

**Conclusions:**

In the presence of other factors, frequent family dinner and vitamin use were associated with lower risk of overweight and household food restriction rules with higher risk. Although such relationships have previously been reported, this investigation is among the first to demonstrate these associations in a low-income, racially-diverse early elementary school population, and suggest potential targets of opportunity within the family context that could reduce child overweight risk in a subgroup of children at elevated risk of obesity.

## Background

Although genetic contributors to childhood obesity and their interactions with the environment cannot be overlooked [1], early environmental influences (during ages two-eight years) in child diet and physical activity have been linked to both current weight status and weight status later in life [2]. Davison and Birch have suggested, based on their longitudinal study of girls, that families can be classified by their patterns of dietary and physical activity behavior, in combination with parent weight status, as obesogenic or non-obesogenic [3]. Subsequent work has suggested that the relationships between child BMI and household environmental factors, such as parental control and feeding styles may be moderated by race/ethnicity, socio-economic status and child age [4-6]. Although substantial evidence from primarily white populations supports the critical role of the family environment and highlights the association between parent weight status and household dietary and physical activity characteristics [6-10], the relationship between household environments of young children and child weight status in racially and ethnically diverse populations has received less attention. In particular, it is unclear whether the relationships between household factors such as family meal frequency and rules and practices around food availability and access are the same across race-ethnic and socio-economic spectrums. Given the higher prevalence of overweight in young non-white and lower socio-economic status children, identification of modifiable household environmental factors is essential to the development of preventive interventions.

Eating dinner together as a family has been associated with fewer sedentary activities, such as TV watching [11], and higher diet quality in children [12]. Likewise, family meals have been positively associated with intakes of fruits, vegetables, dairy products, and several nutrients, and inversely associated with intakes of soft drinks [12-14]. Taveras and colleagues found a lower prevalence of child (ages 9–14) overweight in families that reported family dinner “most days” or “everyday”, as opposed to “never” or “some days” [15]. Some evidence suggests that these beneficial effects may be moderated by race/ethnicity in adolescents [16]. The observation that the cross-sectional associations identified by Taveras et al. did not persist in subsequent *longitudinal* analyses suggests that the relationship between family dinner and child weight status is complex and may be moderated by other factors [15]. For example, it has been suggested that the household *environment* in which meals are eaten, such as allowing the TV to be on or serving prepared foods, may attenuate some of the beneficial effects of family meals [17-20]. Further, the relationship between family meals and obesity has been shown to be moderated by sex in Hispanic and non-Hispanic black children and some studies observe a *positive* relationship between family meal frequency and risk of obesity in Hispanic boys (≤ 12 years) living in low-education households [21]. In sum, further investigation of the associations among family dinner, TV, and child weight status in racially and ethnically diverse populations is warranted.

A considerable body of evidence links restrictive parental practices involving food, particularly snack food, with child overweight [7,22-25]. However, due largely to the cross-sectional nature of most investigations [7,9,26-28] the degree to which parental restriction is a response to child overweight rather than a contributor is not clear. An increased desire for restricted snack foods, compared to unrestricted snack foods was demonstrated in boys and girls ages 3–6 years [27]. Similarly, maternal restriction of foods was associated with decreased ability to self-regulate snack intake, greater snack food consumption and energy intake, and was predictive of child weight status in girls [7]. When this relationship was explored cross-sectionally in a sample of boys and girls, the association was observed only in girls [29]. Indeed, most studies of parental food restriction and child weight status have been limited to Caucasian girls of higher socio-economic status living in two-parent households [28]. The few studies conducted in more diverse populations have found either no association, weak associations, or inverse associations between parental restriction and child overweight [28,30-32]. This represents a critical gap in the literature, inasmuch as the prevalence of overweight is higher in racial and ethnic minorities and in children from lower-socioeconomic status families [31,33].

Likewise, some evidence suggests that household norms surrounding food, child feeding practices and responses to child overweight are different across income levels and race/ethnicity. For example, focus group findings [34] demonstrated a higher propensity to characterize overweight body type schematic images as normal weight among African-American mothers and middle-income white mothers, when compared to low-income white and Hispanic mothers. Likewise, Hispanic mothers reported being more concerned about their children’s health and their eating *enough* than about their body weight, while African-American mothers believed that their children would outgrow overweight and that higher body weight in childhood was healthy [34].

Other studies of household norms and behaviors have found that household food availability and parental consumption of particular foods influence children’s consumption [35], and that child TV viewing is associated with increased risk of being overweight [35-37]. Consumption of sugar-sweetened beverages has also been consistently associated with excess weight in children [38,39].

This paper describes the behaviors and demographic and household characteristics of a group of racially and ethnically diverse, urban-dwelling first through third grade children, and explores cross-sectional associations with weight status. The purpose of this investigation was to further elucidate the complex associations among household and family factors such as family dinner and food rules and practices surrounding food intake and weight status in a low socio-economic, racially/ethnically diverse population.

## Methods

Data used in this analysis were collected in September 2003, at baseline, from children and their families participating in a larger community intervention, Shape Up Somerville: Eat Smart, Play Hard (SUS) [40]. SUS was a 3-year non-randomized, controlled trial involving three communities in Eastern Massachusetts. Details of the intervention are reported elsewhere [40,41]. This study was approved by the Tufts University Institutional Review Board. Assent was obtained from all participants and written informed consent was obtained from all parents.

### Participants

Participants included first through third grade children (mean age 7.6 ± 1.0 years) enrolled in the public school systems of the three communities (30 elementary schools in all) in fall 2003 whose parents provided written informed consent [39]. Of the 5,940 children eligible for the study, consent was obtained for 1721 (29%), all of whom were enrolled. Complete height, weight, and age measures were collected for 1351 children. Reasons for missing data included child absence, child having left the area or the school, child refusal, parent withdrawal, and child disability status precluding measurement. Parent and household data were collected through surveys sent to the households of all 1721 consented children. Among the 963 parents who returned the survey, a significantly higher response rate (p < 0.001) was observed for parents of Caucasian children (73.2%, 478/653), when compared to parents of black (48.1%, 111/231) or Hispanic (53.9%, 145/269) children. Child weight status (categorized by BMI--for-age percentile) did not differ between children whose parents completed the family survey and those who did not. The response rate was 62.2% among parents of normal weight children, 58.3% among parents with overweight children, and 56.2% among parents with obese children. The analyses presented in this paper are restricted to children with complete height/weight/age and parent/household data (n = 820) who were not underweight (97.9%). Not all participants responded to every question.

### Measures

#### Demographic, household, and behavioral measures

In addition to child demographic information, which included race/ethnicity (Caucasian, black, Hispanic, Asian, multiracial, or other), age, grade, and sex, collected at the time informed consent was obtained, a 68-item questionnaire, written in the household language (English, 80.0%; Spanish, 8.4%; Portuguese, 9.5%; and Haitian Creole 2.1%), was mailed to parents/guardians. Parent/guardians were asked to report family socio-demographic information: education (less than high school, high school, some college, or college/graduate school), parent weight and height, and selected household characteristics: frequency of having family dinner (“a lot,” vs. “sometimes”, “rarely”, or “never”), and whether the parent was physically active with the child (“a lot” or “sometimes” vs. “rarely” or “never”). Parents were also asked about household rules restricting both snack intake (yes/no) and kitchen access (yes/no). For these variables, parents were asked, “Are there any rules your child has to follow about snacking?” and “Are there any rules your child has to follow about helping him/herself to food in the kitchen?” Questions pertaining to the child, reported by the parent, included intake of fruits, vegetables and low-fat dairy products (servings per day), consumption of sugar-sweetened beverages (servings per week, not including 100% fruit juice), vitamin/mineral supplement use, total hours and/or minutes per week of screen time (TV, video, computer, and video games), having the TV on during dinner (yes/no), having a TV in the child’s bedroom (yes/no), and child’s involvement in structured physical activities such as team sports or dance lessons over the year (number of sports/lessons per year) [40,41].

#### Child weight status

Baseline measures of height and weight were obtained, in triplicate, without shoes, by qualified, trained staff following recommended procedures for standardized anthropometric measurement of children in school settings, as previously described [40]. In accordance with CDC guidelines, a BMI-for-age below the 5th percentile was considered underweight, a BMI-for-age between the 5th and 85th percentile was considered normal weight, a BMI-for-age at or above the 85th percentile was considered overweight and a BMI-for-age at or above the 95th percentile was considered obese [42].

#### Statistical analysis

Statistical analyses were performed using SPSS statistical software (Version 14.0, SPSS, Inc. 2005, Chicago, IL). Frequency distributions and cross-tabulations of demographic and household variables were examined in relation to normal and overweight categories, with variables of primary empirical interest being frequency of family dinner, household food rules, and race/ethnicity. At the exploratory stage, variables found to be significantly associated with child weight categories (*p value < 0.05)* were included as covariates in logistic regression models. Variables for child sex and age, consumption of sugar-sweetened beverages, and parent education (education level of most highly educated parent was used as a proxy for socioeconomic status) were also included. The initial model contained frequency of family dinner, child race/ethnicity, child sex, child age, parent overweight (one or both parents with calculated BMI > 25), parent education, screen time, two servings or less of sugar-sweetened beverages per week (yes/no), number of low-fat dairy servings per day, and whether the child took any type of vitamin/mineral supplement (yes/no). The sugar-sweetened beverage cut-off represented the lowest quartile of consumption compared to the upper three quartiles. A three-level variable reflecting the use of household rules on snacking and kitchen access rule coded as: zero, one, or two household food rules, was considered in the model.

Finally, based on previous findings in the literature, specific interactions were tested one at a time in the above model. We explored whether the influence of food rules on child weight status was dependent on parent overweight [43,44], as well as the relationship between racial/ethnic group and sex, using child overweight as an outcome [45,46]. Also, based on evidence suggesting that some of the positive effects of family dinner on dietary quality may be diminished by watching TV during dinner [17], the interaction between frequency of family dinner and TV watching during dinner (coded as “a lot” vs. “sometimes”, “rarely” or “never”) was assessed. The results of the models are reported as odds ratios (OR) and their 95% confidence intervals (CI).

All tests were two-sided and level of significance was set at p < 0.05. Independent variables shown to influence the model, based on their statistical significance and impact of their presence or absence in the model on the coefficients of other variables (> 10%), were retained. Variables shown to produce less than 10% change in the other coefficients and non-significant p-values were removed from the final model.

## Results

The prevalence of child overweight (defined as BMI z-score above the 85th percentile for age) in the sample of 820 children was 38.0%. Prevalence was 35.3% for girls and 40.8% for boys. Table 1 displays demographic variables by child weight status (normal weight vs. overweight). Weight status differed by racial/ethnic group (χ^2^ = 16.04, p = 0.014), with Hispanic children most likely (52.2%) to be in the overweight category. As expected, overweight parents were more likely to have overweight children than were normal weight parents (χ^2^ = 21.37, p < 0.001).

**Table 1:** **Distribution of child and family demographic characteristics by child weight status**^**a **^**in a sample of racially and ethnically diverse first through third grade children**

	Full sample (n = 820)*	**Normal (n = 508) 62.0%**	**Overweight (n = 312) 38.0%**	**χ**^**2**^	**p-value**
**Sex (%)**				2.56	0.063
Female	422 (51.7)	273 (64.7)	149 (35.3)		
Male	395 (48.3)	234 (59.2)	161 (40.8)		
**Race/ethnicity (%)**				16.04	0.014
Caucasian	422 (51.9)	266 (63.0)	156 (37.0)		
Black	97 (11.9)	63 (64.9)	34 (35.1)		
Hispanic	115 (14.1)	55 (47.8)	60 (52.2)		
Asian	40 (4.9)	27 (67.5)	13 (32.5)		
Multi-Ethnic	93 (11.4)	61 (65.6)	32 (34.4)		
Other	46 (5.7)	33 (71.7)	13 (28.3)		
**Maximum parent education (%)**				2.71	0.439
< High School	47 (5.9)	27 (57.4)	20 (42.6)		
High School/GED	265 (33.2)	160 (60.4)	105 (39.6)		
Two Year College	202 (25.3)	122 (60.4)	80 (39.6)		
College or beyond	284 (35.6)	187 (65.8)	97 (34.2)		
**Maximum parent weight category (%)**				21.37	< .001
Underweight	5 (0.7)	5 (100)			
Normal	144 (19.8)	106 (73.6)	38 (26.4)		
Overweight	340 (46.8)	223 (65.3)	118 (34.7)		
Obese	237 (32.6)	125 (52.7)	112 (47.3)		
**Grade level (%)**				1.12	0.571
First Grade	319 (39.0)	191 (59.9)	128 (40.1)		
Second Grade	248 (30.4)	156 (62.9)	92 (37.1)		
Third Grade	250 (30.6)	160 (64.0)	90 (36.0)		

^a^Overweight defined as BMI z-score above the 85th percentile for age as recommended by the Centers for Disease Control and Prevention [35,47].

*Sample sizes for individual categories vary due to missing data.

### Health behaviors and household characteristics

Of the parent/guardian respondents, 88% were the child’s mother, 10% were the child’s father, and fewer than two percent were the child’s guardian.

Nearly half of the respondents reported that children ate one serving or fewer of vegetables and one serving or fewer of fruits per day. Mean daily intake was 1.55 (±0.92) servings for vegetables and 1.66 (±0.93) servings for fruits (Table 2). Additionally, 72% (489/677) reported that their child consumed more than two sugar-sweetened beverages per week. Total screen time, including TV, video games, DVD’s, and computers averaged 1404 (±642) minutes per week, or 3.3 hours per day. Nearly 50% of parents reported that their child had a TV in his or her bedroom and almost 40% reported that the child ate dinner with the TV on “a lot” or “sometimes”.

**Table 2:** **Distribution of health behaviors by child weight status**^**a **^**in a sample of racially and ethnically diverse first through third grade children**

	**Full sample (n = 820)**	**Normal (n = 508)**	**Overweight (n = 312)**	**χ**^**2**^	**p-Value**
Sweet beverages/week, n (%)*				4.51	.212
0-2	188 (27.8)	128 (68.1)	60 (31.9)		
3-5	161 (23.8)	101 (62.7)	60 (37.3)		
6-8	157 (23.2)	97 (61.8)	60 (38.2)		
≥9	171 (25.3)	98 (57.3)	73 (42.7)		
Snack rule, n (%)**	591 (74.6)	350 (71.0)	241 (80.6)	9.07	.002
Kitchen access rule, n (%) **	570 (71.4)	338 (68.0)	232 (77.1)	7.55	.017
% Snack + Kitchen Rule, n (%)**	489 (62.0)	285 (58.0)	204 (68.5)	8.53	.002
Family dinner, n (%)*				18.97	<.001
A lot	597 (74.3)	396 (79.5)	201 (65.7)		
Sometimes/rarely/never	207 (25.7)	102 (20.5)	105 (34.3)		
% TV in child’s bedroom**	399 (49.8)	244 (48.9)	155 (51.3)	.443	.277
% TV on during dinner, n (%)*				0.23	.351
A lot/sometimes	323 (39.8)	204 (40.5)	119 (38.8)		
Rarely/never	488 (60.2)	300 (59.5)	188 (61.2)		
% Parent physically active w/ Child, n (%)*				1.65	.120
A lot/sometimes	700 (87.0)	439 (88.2)	261 (85.0)		
Rarely/never	105 (13.0)	59 (11.8)	46 (15.0)		
% Children take vitamins, n (%)**	353 (43.6)	242 (48.2)	111 (36.0)	11.50	.001
**Vegetable servings/day**^+^**	1.55 (0.9)	1.55 (0.9)	1.54 (0.9)		.835
**Fruit servings/day**^+^**	1.66 (0.9)	1.67 (0.9)	1.63 (0.9)		.556
**Dairy servings/day**^+^**	2.67 (1.1)	2.74 (1.1)	2.57 (1.1)		.026
**Snack servings/day**^+^**	1.80 (1.0)	1.84 (1.0)	1.71 (0.9)		.082
**Screen time**^+^** (Minutes/week)	1404 (642)	1360 (636)	1476 (646)		.012
**Sports**^+^** (Number/Year)	2.37 (2.4)	2.41 (2.4)	2.29 (2.3)		.490

^a^Overweight defined as BMI z-score above the 85th percentile for age as recommended by the Centers for Disease Control and Prevention [42].

*= Column percent, difference assessed by χ^2^.

**= Within group percentage, differences assessed by ANOVA.

^+^Mean (±standard deviation).

In unadjusted analyses, patterns for several health behaviors and household characteristics differed by weight status (Table 2). Households with children in the overweight category were more likely to have rules related to snacking (χ^2^ = 9.07, p = 0.002) and about whether or not children were allowed to help themselves to food in the kitchen (χ^2^ = 7.55, p = 0.017) than those with children in the normal weight category. Parents/guardians in households with overweight children reported lower frequency of having family dinner “a lot” than children in the normal category (χ^2^ = 18.97, p < 0.001). There was no difference between the two weight categories in parent/guardian reports of frequency of being physically active with their children. Overweight children were less likely to take vitamin/mineral supplements than normal weight children (χ^2^ = 11.5, p = 0.001). Overweight children had significantly (p = 0.012) more minutes of screen time per week (1476 ± 646) than children in the normal weight category (1360 ± 636 minutes). The number of daily servings of low-fat dairy was higher for normal weight than for overweight children (2.74 vs. 2.57, p = .026), whereas the number of fruits, vegetables, and snacks per day did not vary significantly between groups (Table 2).

Table 3 shows the results of the final logistic regression model predicting the presence of child overweight compared to normal weight. Controlling for the other variables in the model, the likelihood of overweight was higher in Hispanic than Caucasian children (OR = 2.36; CI 1.35 to 4.12). Children in households with one food rule were more likely to be overweight than those in households with no food rules (OR = 2.61; CI 1.33 to 5.09); having two food rules increased the odds ratio for overweight further (3.53; CI, 1.96 to 6.35). Having dinner as a family frequently (reported as “a lot”) and reported child use of vitamin/mineral supplements were associated with a lower likelihood of overweight (OR = 0.47; CI 0.31 to 0.71) and (OR = 0.54; CI, 0.37 to 0.78), respectively. Child sex, child age, number of sugar-sweetened beverages consumed per week, parent overweight and parent education were not significantly related to child overweight in this model.

**Table 3:** Odds of overweight in a sample of racially and ethnically diverse first through third grade children

**Independent variables**	**Beta coefficient**	**SE**	**Odds ratio**	**(95% Cl)**
**Family dinner**				
“A lot”	-.763	.211	.466	(0.31-0.71)
Sometimes/rarely/never	--	--	1.00	Reference
**Number of food rules**				
0 Food rules	--	--	1.00	Reference
1 Food rule	.958	.342	2.61	(1.33-5.09)
2 Food rules	1.26	.300	3.53	(1.96-6.35)
**Racial/ethnic group**				
Caucasian	--	--	1.00	Reference
Black	.048	.306	1.02	(0.58-1.91)
Hispanic	.857	.285	2.36	(1.35-4.12)
Asian	-.103	.433	.902	(0.39-2.11)
Multi	.077	.304	1.08	(0.59-1.96)
Other	-.350	.482	.705	(0.28-1.81)
**Vitamin/mineral supplement use**	-.621	-.192	.538	(0.37-0.78)

Adjusted for: child age, sex, sugar-sweetened beverage consumption, parent overweight, parent education.

No evidence for interactions between sex and racial/ethnic group, parent overweight and food rules, or family dinner and TV viewing during dinner was evident (p-values for all interaction terms exceeded 0.05). Inclusion of each set of interaction terms had negligible effects on the other covariates.

## Discussion

This study is among the first to find that frequent family dinners are associated with lower risk of overweight and that household food rules are associated with higher risk in a socio-economically and racial/ethnically diverse early elementary school population. Specifically, the analyses highlight the cross-sectional relationships between the household practices of family dinners and food rule-setting in relation to child overweight in a multi-racial/ethnic, urban sample of early elementary school children. The influences were evident after adjusting for a wide variety of demographic and behavioral factors. These findings extend previously documented observations regarding the importance of children’s environments, particularly their household environments and parent/caregiver influences, in association with weight status in Caucasian families to racially and ethnically diverse families [3,13,48].

The finding that children whose parents reported having dinner with them frequently were less likely to be overweight than those whose parents reported having dinner with them infrequently or never, is consistent with some, but not all, published research [13-15]. Having the TV on during dinner was not associated with weight status in our sample and we saw no evidence that having the TV on during dinner influenced the association between family dinner and child overweight. Some evidence suggests that, at least for adolescents, the inverse association between family meals and overweight is restricted to non-Hispanic Caucasians [16]. This cross-sectional study demonstrates that this association occurs in other racial/ethnic groups. It should be noted that we assessed only the relationship between TV watching during dinner and weight status. Previous studies have emphasized dietary *quality* in relation to this practice. While family meals without the TV have consistently been associated with higher dietary quality, at least one study demonstrated that even with TV use, family meals provide a dietary quality advantage for children when compared to absence family meals [49].

The finding of greater odds of overweight in children from households with at least one rule restricting food access, and still greater odds in the presence of two such rules was also consistent with previous findings [7,26,27]. However, because temporality cannot be determined from cross-sectional data, we are not able to determine whether household food rules restricting snacking and kitchen access precipitated overweight or whether such rules were established by parents in response to their child’s weight status. Others have suggested that relationships between parental rule-setting about food intake and children’s weight status are complex and not unidirectional [9,29,50]. Most studies of food rules and parental restriction have been limited to Caucasian and higher socioeconomic populations [51,52]. These findings document this association in a racially/ethnically diverse population.

Use of vitamin/mineral supplements was found to vary by weight category and thus was tested in the regression model. It may be that vitamin/mineral supplement use is a proxy for a consistent, organized household or healthy behaviors resulting from overall adaptive family functioning [53]. The concept of household order has been associated with better outcomes in families for other pediatric conditions [54-56], but has been less frequently explored in the context of childhood obesity [57,58]. Interestingly, Joyce and colleagues [59] demonstrated that “parenting chaos”, or the degree of inconsistency and unpredictability in parenting routines in the context of eating [60], may moderate the relationships among food restriction, disinhibited eating and child BMI. Specifically, the association between food restriction and disinhibited eating was stronger in households with higher levels of parent chaos [59].

Having frequent family dinners may also be related to household order [61]. It is also plausible that use of vitamin/mineral supplements and having frequent family dinners may correlate with socio-economic status (SES). That low SES may be associated with a poorer home environment overall and thereby an increased risk for obesity has been previously observed [62,63]. However, parental education, an SES indicator, was accounted for in this present study. Overall, these findings suggest the need for further investigation and, as Fiese and colleagues indicate the importance of investigating household and family routines in the broader context of social, economic and cultural factors [64].

Indeed, differences in *context* may explain discrepancies seen across studies in associations between childhood weight status and the household and family factors discussed above. For instance, child feeding practices have been shown to vary across both socioeconomic status and racial/ethnic groups [65] and some have suggested that *cumulative* effects of neighborhood and family level factors must be considered when explaining environmental risk factors for childhood obesity [64]. Gaps remain in fully describing these associations.

This study had several strengths. This investigation lends to the knowledge base in this area as it was conducted in an ethnically diverse population, assessed a range of socio-demographic variables, and explored a wide variety of behavioral and household issues. All questionnaires and study materials were translated into parents’ native languages to support inclusion of a more diverse sample of respondents than has typically been included in similar studies. Finally, sample size was sufficient to build a model that supported analysis of a substantial number of variables simultaneously.

Several limitations are noteworthy. Most importantly, the cross-sectional design precludes determination of causality. Also, this study, like others [15] used qualitative descriptors of characteristics that are poorly suited for direct quantitative interpretation. For example, physical activity was assessed via questions about involvement in *organized* physical activity, such as team sports or swimming lessons. While this approach yields information about the type, frequency, and consistency with which children in the sample engaged in physical activity, it fails to capture non-organized physical activities, such as riding a bicycle or playing neighborhood basketball. This discrepancy may explain the lack of an observed effect of physical activity on weight status. Additionally, all information from parent questionnaires was self-reported and, as such, may be subject to both error and bias. Further, the dichotomous (yes/no) response choices for the household food rule questions do not fully capture nuances that are likely to exist in the area of household food restriction. For instance, food rules may be time or context-specific, permanent or temporary, and may represent a wide range in terms of degree of restrictiveness. Lastly, the response rate from parents of racial/ethnic minority children was significantly lower than for parents of Caucasian children. This may have influenced the internal validity and generalizability of results.

## Conclusions

This study adds to the body of evidence supporting an inverse association between frequency of family meals and child overweight, and to the literature suggesting a positive association between restrictive parental food rules and child overweight. To date, research on the role of household and family factors and child weight status in diverse and low socioeconomic status populations has been limited and to some degree inconclusive. Given the disproportionate prevalence of pediatric obesity in these populations, large scale, prospective, hypothesis-driven studies using valid, comprehensive instruments [66] are needed to explore the interaction of family and household characteristics, on the development of children’s eating and physical activity habits and the subsequent effects on weight status. Elucidating specific mechanisms through which parents exert influence, such as rule-setting, modeling, shaping attitudes and preferences, or influencing the development of self-regulation and how these interact with parent weight status and other environmental contexts may be of particular importance. Future studies should also consider looking at a broad age range - from preschool through young adulthood - to determine how these effects may change over time.

## Competing interests

Sonya Irish Hauser: reports no competing interests.

Christina D. Economos: reports no competing interests.

Miriam Nelson: reports no competing interests.

Jeanne P. Goldberg: reports no competing interests.

Raymond Hyatt: reports no competing interests.

Elena N. Naumova: reports no competing interests.

Sarah E. Anderson: reports no competing interests.

Aviva Must: reports no competing interests.

We certify that this manuscript is original work and has not been published elsewhere. The authors do not have any conflicts of interest, financial or otherwise. All authors listed have read the manuscript, agree that the manuscript is ready for submission to a journal and are willing to accept responsibility for the manuscript’s contents.

## Authors’ contributions

SIH performed data analysis and interpretation and drafted the manuscript. CDE was the principal investigator for the larger intervention from which data for this current analysis was drawn and assisted in drafting and editing the manuscript. MN participated in design of the larger intervention contributed to manuscript editing JPG participated in design of the larger intervention contributed to manuscript editing RH assisted in data analysis and manuscript editing ENN participated in design of the larger intervention contributed to manuscript editing. SEA assisted in data analysis, interpretation and manuscript editing AM participated in design of the larger intervention, interpretation of analyses, and contributed to manuscript conceptualization and writing. All authors read and approved the final manuscript.

## Pre-publication history

The pre-publication history for this paper can be accessed here:

http://www.biomedcentral.com/1471-2431/14/167/prepub
